# Supplementary material for: The Small RNA Universe of Capitella teleta
Source: Front Mol Biosci. 2022 Feb 25;9:802814. doi: 10.3389/fmolb.2022.802814 (PMC8915122; doi:10.3389/fmolb.2022.802814)
Supplement: Supplementary file 1 [file DataSheet1.ZIP › Supplement/known/CAPTEscaffold_70_6928.pdf]

Provisional ID	:	CAPTEscaffold_70_6928
Score total	:	22447.9
Score for star read(s)	:	3.9
Score for read counts	:	22438.5
Score for mfe	:	0.8
Score for randfold	:	1.6
Score for cons. seed	:	3
Total read count	:	44024
Mature read count	:	36131
Loop read count	:	0
Star read count	:	7893

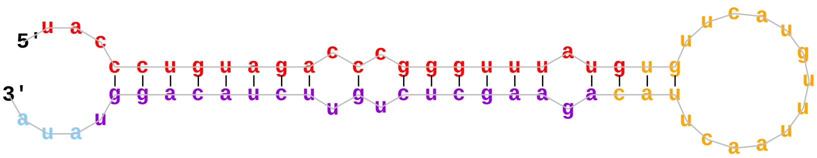

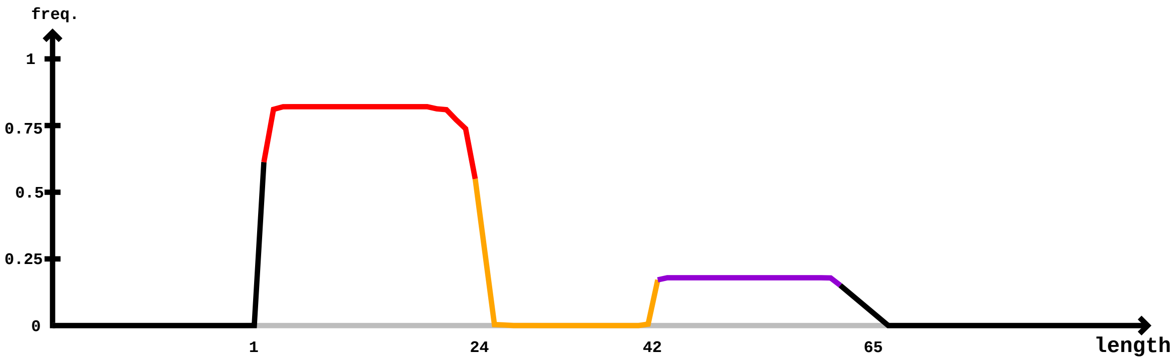

Mature **Star**

5'- ucccguucacauuccgcauguacccuguagacccggguuuauguguucauguuuaacuuacagaagcucuguucuacagguauauuuugaaugugcuaacaccuaauccguu -3'	obs		
ucccguucacauuccgcauguacccuguagacccggguuuauguguucauguuuaacuuacagaagcucuguucuacagguauauuuugaaugugcuaacaccuaauccguu	exp		
ucccguucacauuccgcauguacccuguagacccggguuuauguguucauguuuaacuuacagaagcucuguucuacagguauauuuugaaugugcuaacaccuaauccguu	known		
....((((((((((...(((((((.((((((.(.((((((.((((............)))).)))))).).)))))))))))))...)))))))..))).............	reads	mm	sample
...................Cuacccuguagacccggguuu..	1	1	seq
...................Cuacccuguagacccggguuuau..	2	1	seq
...................Cuacccuguagacccggguuuaugu..	3	1	seq
....................uacccuguagacccgggu..	142	0	seq
....................uacccuguagacccgggA..	211	1	seq
....................uacccuguagacccgggG..	1	1	seq
....................uacccuguagacccggguu...	133	0	seq
....................uacccuguagacccgggAu...	1	1	seq
....................uacccuguagaccAggguu...	1	1	seq
....................uacccuguagacccggguuA..	1	1	seq
....................uacccuguagacccAgguuu..	2	1	seq
....................uacccuguagaAccggguuu..	1	1	seq
....................uacccCguagacccggguuu..	1	1	seq
....................Aacccuguagacccggguuu..	4	1	seq
....................uacccuguagaccUggguuu..	2	1	seq
....................uacccuguagacccgAguuu..	3	1	seq
....................uaccUuguagacccggguuu..	2	1	seq
....................uacccuguagacUcggguuu..	2	1	seq
....................uacccuguagacccgggGuu..	1	1	seq
....................uacccuguagacccggguuu..	1573	0	seq
....................uacccuguagacAcggguuu..	5	1	seq
....................uacccuguagCcccggguuua...	1	1	seq
....................uacccuguagaAccggguuua...	1	1	seq
....................uacccugCagacccggguuua...	1	1	seq
....................uacccuguagacccgggAuua...	1	1	seq
....................uacccuguagaccUggguuua...	1	1	seq
....................uaccUuguagacccggguuua...	2	1	seq
....................uacccuguagacccggguuuU...	5	1	seq
....................uacccuguagacccAgguuua...	1	1	seq
....................uacccuguagaccAggguuua...	1	1	seq
....................uacccuguagacccggguuua...	944	0	seq

Mature Star

ucccguucacauuccgcauguacccuguagacccggguuuauguguucauguuuaacuuacagaagcucuguucuacagguauauuuugaaugugcuaacaccuaauccguu			
....................Cacccuguagacccggguuua...	2	1	seq
....................uacccuguagacccggguuAa...	1	1	seq
....................uacccuguagacccggguuuG...	1	1	seq
....................uacccuguagacccAgguuuau..	14	1	seq
....................uacccuguagacccggguuuaA..	7	1	seq
....................uacccuguagacccgCguuuau..	2	1	seq
....................uaccGuguagacccggguuuau..	2	1	seq
....................uUcccuguagacccggguuuau..	1	1	seq
....................uacccugCagacccggguuuau..	2	1	seq
....................uacccuguagacccgggAuuau..	6	1	seq
....................uacccuCuagacccggguuuau..	1	1	seq
....................uacAcuguagacccggguuuau..	6	1	seq
....................uaccAuguagacccggguuuau..	4	1	seq
....................uacccuguagacUcggguuuau..	5	1	seq
....................uacccuguagacccggguuuaC..	3	1	seq
....................uaccUuguagacccggguuuau..	8	1	seq
....................Aacccuguagacccggguuuau..	21	1	seq
....................uacccuguaAacccggguuuau..	1	1	seq
....................uacccuguagacccggguuuau..	7562	0	seq
....................uacccuguagacccCgguuuau..	2	1	seq
....................uacccuguagacccggguuuGu..	1	1	seq
....................uacccuguagacGcggguuuau..	1	1	seq
....................uacccuguagacccgAguuuau..	3	1	seq
....................uacccuguGgacccggguuuau..	1	1	seq
....................uacccuguagaUccggguuuau..	2	1	seq
....................uacGcuguagacccggguuuau..	1	1	seq
....................uacccuguagaAccggguuuau..	14	1	seq
....................uacccuguagaGccggguuuau..	1	1	seq
....................uacccuguagaccUggguuuau..	3	1	seq
....................uacccuguagUcccggguuuau..	1	1	seq
....................uacccuguagacAcggguuuau..	2	1	seq
....................uacccuAuagacccggguuuau..	1	1	seq
....................Nacccuguagacccggguuuau..	2	1	seq
....................uacccuguagacccgggGuuau..	6	1	seq
....................uacccuguagacccggguGuau..	1	1	seq
....................uacUcuguagacccggguuuau..	1	1	seq
....................uacccAguagacccggguuuau..	1	1	seq
....................uacccCguagacccggguuuau..	3	1	seq
....................Cacccuguagacccggguuuau..	2	1	seq
....................uacccuguagacccggguuuCu..	1	1	seq
....................uacccugAagacccggguuuaug...	13	1	seq
....................uacccuguagacccggguuuaGg...	1	1	seq
....................uacccuguagaAccggguuuaug...	11	1	seq
....................uacccuguaUacccggguuuaug...	1	1	seq
....................uacccuguagaccGggguuuaug...	1	1	seq
....................uacccuguagacccgggCuuaug...	1	1	seq
....................uacccuguagacccggguuuauU...	57	1	seq
....................uacccugCagacccggguuuaug...	1	1	seq
....................uacccuguagacccggguuuauC...	3	1	seq
....................uacccuguagacGcggguuuaug...	2	1	seq
....................uacccuguagaUccggguuuaug...	3	1	seq
....................uGcccuguagacccggguuuaug...	1	1	seq
....................uacccuguagacccggUuuuaug...	5	1	seq
....................uaAccuguagacccggguuuaug...	4	1	seq
....................uacccuguagacccggguuuaug...	13310	0	seq
....................uaccGuguagacccggguuuaug...	1	1	seq
....................uaccUuguagacccggguuuaug...	8	1	seq
....................uacccuguagacccAgguuuaug...	21	1	seq
....................uacccuguagacccggguuuGug...	1	1	seq
....................uacccuguagaccUggguuuaug...	10	1	seq
....................uacccuguagacccggguuGaug...	1	1	seq
....................uacccuAuagacccggguuuaug...	3	1	seq
....................uacccuguagacAcggguuuaug...	17	1	seq
....................uacccuguagacccggguGuaug...	2	1	seq
....................uacccuguGgacccggguuuaug...	3	1	seq
....................uacccuguagacccggAuuuaug...	1	1	seq
....................uacUcuguagacccggguuuaug...	6	1	seq
....................uacccuguagacccgggGuuaug...	12	1	seq
....................Nacccuguagacccggguuuaug...	4	1	seq
....................uacccuguagaGccggguuuaug...	1	1	seq

Mature **Star**

ucccguucacauuccgcaugacccuguagacccggguuuauguguucauguuuaacuuacagaagcucuguucuacagguauauuuugaaugugcuaacaccuaauccguu

....................uacccuguagacUcggguuuaug...	4	1	seq
....................uacccuguagacccggguCuaug...	1	1	seq
....................uacAcuguagacccggguuuaug...	1	1	seq
....................uacccuguagacccgCguuuaug...	1	1	seq
....................uacccuUuagacccggguuuaug...	1	1	seq
....................uacccuguagacccgAguuuaug...	9	1	seq
....................uacccuguaAacccggguuuaug...	4	1	seq
....................uacccuguaCacccggguuuaug...	2	1	seq
....................uaUccuguagacccggguuuaug...	2	1	seq
....................uacccuguagacccggguuuaCg...	1	1	seq
....................uacccuguagacccggguuuaAg...	4	1	seq
....................uacccuguagacccgUguuuaug...	2	1	seq
....................uacccuguagacccggCuuuaug...	1	1	seq
....................uacccuguagaccAggguuuaug...	1	1	seq
....................uacccuguagacccggguuuUug...	1	1	seq
....................Aacccuguagacccggguuuaug...	28	1	seq
....................uacccuguagGcccggguuuaug...	3	1	seq
....................Cacccuguagacccggguuuaug...	3	1	seq
....................uacccuguagacccggguAuaug...	6	1	seq
....................uacccuguagacccggguuuauA...	302	1	seq
....................uacccAguagacccggguuuaug...	8	1	seq
....................uacccuguagacccCgguuuaug...	1	1	seq
....................uacccuguagacccgggAuuaug...	9	1	seq
....................uacccuguagacccggguuCaug...	1	1	seq
....................uacccuguagacccggguuAaug...	2	1	seq
....................Gacccuguagacccggguuuaug...	3	1	seq
....................uacccuguagacccUgguuuaug...	1	1	seq
....................uacGcuguagacccggguuuaug...	1	1	seq
....................uacccuguagacccgggGuuaugu..	1	1	seq
....................uacccuguagacccggguuuauUu..	2	1	seq
....................uacccGguagacccggguuuaugu..	1	1	seq
....................uacccuguagacccggguuuaugG..	21	1	seq
....................uacccuguagacccUgguuuaugu..	1	1	seq
....................uGcccuguagacccggguuuaugu..	1	1	seq
....................uacccuguagacUcggguuuaugu..	2	1	seq
....................uacccuguagacccggguAuaugu..	1	1	seq
....................uacccuguagacAcggguuuaugu..	2	1	seq
....................uacccuguagacccgggAuuaugu..	1	1	seq
....................uacccuguagacccgAguuuaugu..	2	1	seq
....................uacccuguagacccggAuuuaugu..	1	1	seq
....................uacccuguagacccggguuuaugu..	1005	0	seq
....................uacccuguagacccggguuuaugA..	1255	1	seq
....................uacccuguagacccggguuuaugC..	5	1	seq
....................uacccuguagacccggguuuauAu..	2	1	seq
....................uacccuguagacccggguuuaugug...	3	0	seq
....................uacccuguagacccggguuuauguU...	9	1	seq
....................uacccuguagacccggguuuaugugu..	8	0	seq
....................uacccuguagacccggguuuauguCu..	1	1	seq
....................uacccuguagacccggguuuaugugG..	1	1	seq
....................uacccuguagacccggguuuauguguuca...	2	0	seq
....................uacccuguagacccggguuuauguguucaug...	1	0	seq
.....................acccuguagacccggguu...	3	0	seq
.....................Gcccuguagacccggguuu..	1	1	seq
.....................acccuguagacccggguuu..	50	0	seq
.....................acccuguagacccAgguuu..	1	1	seq
.....................Gcccuguagacccggguuua...	6	1	seq
.....................acccAguagacccggguuua...	2	1	seq
.....................acccuguagacccggguuua...	501	0	seq
.....................acccuguagaccUggguuua...	1	1	seq
.....................acccuguagaUccggguuua...	1	1	seq
.....................acccuguagacccggguAua...	1	1	seq
.....................accAuguagacccggguuua...	1	1	seq
.....................acccuguagacccggguuuaA..	2	1	seq
.....................acccuguagacccggguuCau..	1	1	seq
.....................acccuguagacccgAguuuau..	1	1	seq
.....................acccuguagacccggguuAau..	1	1	seq
.....................acccuguagacccggCuuuau..	1	1	seq
.....................Gcccuguagacccggguuuau..	6	1	seq
.....................acccAguagacccggguuuau..	7	1	seq
.....................aUccuguagacccggguuuau..	1	1	seq

```
                     Mature                                   Star
ucccguucacauuccgcauguacccuguagacccggguuuauguguucauguuuaacuuacagaagcucuguucuacagguauauuuugaaugugcuaacaccuaauccguu
.....................acccuguagacccggguuuau......................................................................    571    0    seq
.....................acccuguagaccAggguuuau......................................................................    1      1    seq
.....................acccuguagacccggguuuauA.....................................................................    14     1    seq
.....................acccuguagacAcggguuuaug.....................................................................    6      1    seq
.....................accUuguagacccggguuuaug.....................................................................    2      1    seq
.....................acccuguagacccggguuuauU.....................................................................    2      1    seq
.....................acccugAagacccggguuuaug.....................................................................    2      1    seq
.....................acccuguGgacccggguuuaug.....................................................................    1      1    seq
.....................acccuguagaccUggguuuaug.....................................................................    3      1    seq
.....................acccuguagacccggguuuaug.....................................................................    2925   0    seq
.....................acccuguagacccggguuuaGg.....................................................................    1      1    seq
.....................acAcuguagacccggguuuaug.....................................................................    1      1    seq
.....................acccAguagacccggguuuaug.....................................................................    15     1    seq
.....................acccuguagacccggguGuaug.....................................................................    1      1    seq
.....................acccuguagacccgAguuuaug.....................................................................    3      1    seq
.....................aAccuguagacccggguuuaug.....................................................................    1      1    seq
.....................aUccuguagacccggguuuaug.....................................................................    1      1    seq
.....................acccuguagacccgggAuuaug.....................................................................    2      1    seq
.....................Gcccuguagacccggguuuaug.....................................................................    29     1    seq
.....................acccuguagacccggguAuaug.....................................................................    1      1    seq
.....................acccuguagacccAgguuuaug.....................................................................    6      1    seq
.....................acccuguagacccggguuCaug.....................................................................    1      1    seq
.....................acccuguagaGccggguuuaug.....................................................................    1      1    seq
.....................Ncccuguagacccggguuuaug.....................................................................    1      1    seq
.....................acccuguagacccgUguuuaug.....................................................................    1      1    seq
.....................acUcuguagacccggguuuaug.....................................................................    4      1    seq
.....................acccuguagacccggguuGaug.....................................................................    1      1    seq
.....................accGuguagacccggguuuaug.....................................................................    1      1    seq
.....................acccuguagacccggguuuaAg.....................................................................    3      1    seq
.....................acccuguagaccGggguuuaug.....................................................................    2      1    seq
.....................acccuCuagacccggguuuaug.....................................................................    1      1    seq
.....................acccugCagacccggguuuaug.....................................................................    1      1    seq
.....................acccuguagacUcggguuuaug.....................................................................    1      1    seq
.....................acccuguagacUcggguuuaugu....................................................................    3      1    seq
.....................acccAguagacccggguuuaugu....................................................................    11     1    seq
.....................acccugAagacccggguuuaugu....................................................................    5      1    seq
.....................acccuguagaccUggguuuaugu....................................................................    3      1    seq
.....................acccuguagacccggguuuGugu....................................................................    2      1    seq
.....................acccuguagacccCgguuuaugu....................................................................    1      1    seq
.....................acccuguGgacccggguuuaugu....................................................................    1      1    seq
.....................acccuguagacccgggAuuaugu....................................................................    1      1    seq
.....................acccuguagacccAgguuuaugu....................................................................    5      1    seq
.....................acAcuguagacccggguuuaugu....................................................................    2      1    seq
.....................acccuguagacccggguuuaugA....................................................................    88     1    seq
.....................acccuguagaAccggguuuaugu....................................................................    1      1    seq
.....................Ucccuguagacccggguuuaugu....................................................................    1      1    seq
.....................acccuguagacccggguuuaugC....................................................................    1      1    seq
.....................acccuguagacccgAguuuaugu....................................................................    2      1    seq
.....................accUuguagacccggguuuaugu....................................................................    2      1    seq
.....................acccuguagaccGggguuuaugu....................................................................    1      1    seq
.....................acccuguaUacccggguuuaugu....................................................................    1      1    seq
.....................acccuguagacccggguuuaugu....................................................................    4195   0    seq
.....................acccGguagacccggguuuaugu....................................................................    2      1    seq
.....................acccuguagaUccggguuuaugu....................................................................    1      1    seq
.....................acccuguagacAcggguuuaugu....................................................................    6      1    seq
.....................acccuguagGcccggguuuaugu....................................................................    1      1    seq
.....................Gcccuguagacccggguuuaugu....................................................................    35     1    seq
.....................acccuguagacccggguuuaugG....................................................................    3      1    seq
.....................acccuguagaccAggguuuaugu....................................................................    1      1    seq
.....................Ncccuguagacccggguuuaugu....................................................................    1      1    seq
.....................acUcuguagacccggguuuaugu....................................................................    2      1    seq
.....................acccuguagacccgCguuuaugu....................................................................    1      1    seq
.....................acccuguagacccggguuuaGgu....................................................................    1      1    seq
.....................acccuguagacccggguuuaugug...................................................................    7      0    seq
.....................acccuguagacccggguuuauguU...................................................................    23     1    seq
.....................acccuguagacccggguuuaugAg...................................................................    1      1    seq
.....................acccuguagacccggguuuauguA...................................................................    21     1    seq
.....................acccuguagacccggguuuaugugG..................................................................    13     1    seq
.....................acccuguagacccggguuuaugugA..................................................................    1      1    seq
.....................Gcccuguagacccggguuuaugugu..................................................................    1      1    seq
```

Mature **Star**

```
ucccguucacauuccgcauguacccuguagacccggguuuauguguucauguuuaacuuacagaagcucuguucuacagguauauuuugaaugugcuaacaccuaauccguu
.....................acccuguagacccggguuuauguUu..................................................................	2	1	seq
.....................acccuguagacccggguuuaugugu..................................................................	64	0	seq
.....................acccuguagacccggguuuauguguu.................................................................	1	0	seq
.....................acccuguagacccgAguuuauguguu.................................................................	1	1	seq
......................cccuguagacccggguuua.......................................................................	14	0	seq
......................cccuguagacccggguuuau......................................................................	6	0	seq
......................cccuguagacccAgguuuaug.....................................................................	1	1	seq
......................cccuguagacccggguuuaug.....................................................................	77	0	seq
......................cccuguagacccggguuuaugA....................................................................	2	1	seq
......................cccuguagacccggguuuUugu....................................................................	1	1	seq
......................cccuguagacccggguuuaugu....................................................................	322	0	seq
......................ccUuguagacccggguuuaugu....................................................................	1	1	seq
......................Uccuguagacccggguuuaugu....................................................................	1	1	seq
......................cccuguagacccggguuAaugu....................................................................	1	1	seq
......................cccuguagacAcggguuuaugu....................................................................	1	1	seq
......................cccuguagacccgAguuuaugu....................................................................	1	1	seq
......................ccAuguagacccggguuuaugu....................................................................	1	1	seq
......................cccuguagacccggguuuaugugu..................................................................	2	0	seq
.......................ccuguagacccggguuuaug.....................................................................	1	0	seq
.......................ccuguagacccggguuuaugu....................................................................	8	0	seq
...........................................................acagaagcucuguucuacaggu...............................	1	0	seq
............................................................cagaagcucuguucuacag.................................	3	0	seq
............................................................cagaagcucuguucuacagg................................	53	0	seq
............................................................Uagaagcucuguucuacaggu...............................	1	1	seq
............................................................cagaagcucuguucuacaggu...............................	119	0	seq
............................................................cagaagcucuguucuacaggG...............................	1	1	seq
............................................................cagaagcucuguucuacaggA...............................	1	1	seq
............................................................cagaagcucuguucuacaggua..............................	14	0	seq
............................................................Uagaagcucuguucuacaggua..............................	1	1	seq
............................................................cagaagcucuguucuacagguU..............................	2	1	seq
............................................................cagaagcucuguucuacagguaC.............................	4	1	seq
............................................................cagaagcucuguucuacagguau.............................	38	0	seq
............................................................Nagaagcucuguucuacagguau.............................	1	1	seq
.............................................................agaagcucuguucuacag.................................	27	0	seq
.............................................................agaagcucugAucuacagg................................	2	1	seq
.............................................................agaagcucGguucuacagg................................	1	1	seq
.............................................................agaagcucAguucuacagg................................	1	1	seq
.............................................................agaagAucuguucuacagg................................	1	1	seq
.............................................................agaagcuAuguucuacagg................................	1	1	seq
.............................................................agaagcucuguucuacagA................................	17	1	seq
.............................................................agaagcucuguucuacGgg................................	1	1	seq
.............................................................agaagcuUuguucuacagg................................	1	1	seq
.............................................................agaagcucuguAcuacagg................................	1	1	seq
.............................................................agaagcucuguucuacagg................................	1141	0	seq
.............................................................agaagcucugCucuacagg................................	1	1	seq
.............................................................agaagcucuguucCacagg................................	1	1	seq
.............................................................agaagAucuguucuacaggu...............................	3	1	seq
.............................................................agaagcuAuguucuacaggu...............................	2	1	seq
.............................................................agaagcucuguucuacGggu...............................	2	1	seq
.............................................................agaagcucuguuAuacaggu...............................	1	1	seq
.............................................................agaagcucuguucuacaggA...............................	17	1	seq
.............................................................agGagcucuguucuacaggu...............................	3	1	seq
.............................................................agaUgcucuguucuacaggu...............................	1	1	seq
.............................................................agaagcucuguucAacaggu...............................	1	1	seq
.............................................................agaagcucAguucuacaggu...............................	3	1	seq
.............................................................agaagcucuguucuacaggu...............................	3018	0	seq
.............................................................agaagcucugAucuacaggu...............................	1	1	seq
.............................................................aAaagcucuguucuacaggu...............................	1	1	seq
.............................................................agaagcucuguucuacaggG...............................	1	1	seq
.............................................................agaagcucuguucuacaAgu...............................	2	1	seq
.............................................................agaagcucuguucuacaggua..............................	467	0	seq
.............................................................agaagcucuguucuacagguU..............................	6	1	seq
.............................................................agaagcucuguAcuacagguau.............................	1	1	seq
.............................................................agaagcucuguucuacagCuau.............................	1	1	seq
.............................................................agaagcucuguuAuacagguau.............................	2	1	seq
.............................................................agaagcuUuguucuacagguau.............................	1	1	seq
.............................................................agaagcucuguucuacagguaA.............................	49	1	seq
.............................................................agaagcucuguuUuacagguau.............................	1	1	seq
.............................................................agaGgcucuguucuacagguau.............................	1	1	seq
.............................................................agaagcAcuguucuacagguau.............................	2	1	seq
```

Mature Star

```
ucccguucacauuccgcauguacccuguagacccgguuuauguguucauguuuaacuuacagaagcucuguucuacagguauauuuugaaugugcuaacaccuaauccguu
............................................................agaagcucuguucuacagguaC.............................    7      1   seq
............................................................agaagcucuguucuacagguCu.............................    1      1   seq
............................................................agaagcucuguucuacagguaG.............................    1      1   seq
............................................................Ngaagcucuguucuacagguau.............................    1      1   seq
............................................................agaagcucuguucuacagguUu.............................    15     1   seq
............................................................agaagcCcuguucuacagguau.............................    1      1   seq
............................................................agaagcucuguucAacagguau.............................    1      1   seq
............................................................agaagcucuguucCacagguau.............................    1      1   seq
............................................................agaagcucuCuucuacagguau.............................    2      1   seq
............................................................agaagcucuguucuacaggGau.............................    1      1   seq
............................................................agaagcucugAucuacagguau.............................    2      1   seq
............................................................agaagcucuguucuacagguau.............................    2387   0   seq
............................................................agaagcucuguucuacaggAau.............................    1      1   seq
............................................................agaagAucuguucuacagguau.............................    2      1   seq
............................................................agaaCcucuguucuacagguau.............................    1      1   seq
............................................................agaagcucuguucuacagguGu.............................    2      1   seq
............................................................agaagcuAuguucuacagguau.............................    1      1   seq
............................................................agaagcucuguucuacagguauU............................    85     1   seq
............................................................agaagcucuguucuacagguaAa............................    2      1   seq
............................................................agaagcucuguucuacagguauC............................    1      1   seq
............................................................agaagcucuguucuacagguaua............................    6      0   seq
............................................................agaagcucuguucuacagguauUu...........................    3      1   seq
.............................................................gaagcucuguucuacagg................................    4      0   seq
.............................................................gaagcucuguucuacaggu...............................    18     0   seq
.............................................................gaagcucuguucuaUaggu...............................    1      1   seq
.............................................................gaagcucuguucuacaggua..............................    6      0   seq
.............................................................gaagcucuguucuacagguau.............................    131    0   seq
.............................................................gaagcucuguucuacagguUu.............................    1      1   seq
.............................................................gaagcGcuguucuacagguau.............................    1      1   seq
.............................................................Uaagcucuguucuacagguau.............................    1      1   seq
.............................................................Aaagcucuguucuacagguau.............................    1      1   seq
.............................................................gaagcucuguucuacagguauU............................    159    1   seq
.............................................................gaagcucuguucuacagguaua............................    23     0   seq
.............................................................gaagcucuguucuacagguauC............................    1      1   seq
.............................................................gaagcucuguucuacagguauUu...........................    1      1   seq
```
